# Poly-(3-ethyl-3-hydroxymethyl)oxetanes—Synthesis and Adhesive Interactions with Polar Substrates

**DOI:** 10.3390/polym12010222

**Published:** 2020-01-16

**Authors:** Paweł Parzuchowski, Mariusz Ł. Mamiński

**Affiliations:** 1 Faculty of Chemistry, Warsaw University of Technology, 3 Noakowskiego St., 00-664 Warsaw, Poland; pparzuch@ch.pw.edu.pl; 2 Institute of Wood Science and Furniture, Warsaw University of Life Sciences—SGGW, 159 Nowoursynowska St., 02-776 Warsaw, Poland

**Keywords:** polyoxetanes, adhesion, hot melt adhesive

## Abstract

Hyperbranched polyoxetanes are a relatively new class of polymers. These are branched polyethers that are synthesized from oxetanes—four-member cyclic ethers bearing hydroxymethyl groups—via ring-opening polymerization. Four series of polyoxetanes were synthesized from 3-ethyl-3-(hydroxymethyl)oxetane and 1,1,1-tris(hydroxymethyl)propane as a core molecule. Reagents ratios ranged from 1:5 to 1:50, theoretical molar mass ranged from 714 g/mol to 5942 g/mol, and dispersities ranged from 1.77 to 3.75. The morphology of the macromolecules was investigated by a matrix-assisted laser desorption/ionization time of flight technique. The polyoxetanes’ adhesive interactions with polar materials were analyzed and provided results as follows: the work of adhesion was 101–105 mJ/m^2^, the bond-line tensile shear strengths were 0.39–1.32 MPa, and there was a brittle fracture mode within the polymer. The findings confirmed a good adhesion to polar substrates, but further research on polyoxetane modifications toward a reduction of brittleness is necessary.

## 1. Introduction

Hyperbranched polyoxetanes (POXs) are a relatively new class of polymers. The first syntheses were independently reported by Hult’s and Penczek’s groups in 1999 [1,2]. However, co-polymerizations of monomers with both oxetanyl and styryl groups were already reported by Motoi et al. in 1989 [3]. POXs are classified as branched polyethers and are synthesized from oxetanes—four-member cyclic ethers bearing hydroxymethyl groups. In the literature, most reports are focused on the polymerization or co-polymerization of 3-ethyl-3-(hydroxymethyl)oxetane (EHO) [4,5,6,7,8,9,10,11,12,13,14]. The monomer for this process is easily available from trimethylolpropane. The main synthetic approach to polyoxetanes goes via cationic ring-opening polymerization, as an anionic mechanism was found to be less effective due to either a low molar mass (ca. 500 g/mol) or broad dispersities (*M*_w_/*M*_n_ = 4.0–5.5) and a low degree of branching of the products [15,16]. Investigations on the mechanisms of polymerizations revealed that they undergo an active chain end mechanism (ACE) or an activated monomer mechanism (AMM) [17]. The ACE mechanism is more efficient, as cyclization reactions do not occur.

Polyoxetanes are not paid sufficient attention, though their chemical properties like the ease of side chain functionalization or grafting make them versatile and valuable scaffolds in many fields of material science. Reports on the applicability of functionalized polyoxetanes cover, among others, liquid crystals [18], polymer electrolytes with lithium ion coordination abilities [19,20], fluorine-containing hydrophobic materials [21], amphiphilic block copolymers with poly(ethylene oxide) [22], hybrid networks of fluorine-bearing polyoxetane and inorganic phases [23], hybrid fluorine-polyoxetane–polyurethane coatings [24], and hyperbranched polyoxetanes in drug delivery systems [25,26,27]. Wynne and co-workers also used functionalized polyoxetanes to incorporate telechelics in polyurethanes [28,29].

As the number of papers on polyoxetanes is relatively low, the number of works on the adhesive properties of POXs is even lower. Hardly any reports on polyoxetane-based adhesives can be found in the literature.

Jia at al. described a catechol-containing polyoxetane as a platform for an adhesive curable with FeCl_3_ [30]. The investigated adhesives exhibited relatively good strengths on such substrates like metals (3.7–4.9 MPa), poplar wood (2.7 MPa), and glass (2.10 MPa). Further research in that group demonstrated that merging catechol with phosphoric acid and a POX backbone might induce adhesion in humid environments, which may give way to the design of biomimetic adhesives [31]. Reports on photocurable adhesive systems based on epoxides and oxetanes can be also found in the literature [32,33].

In 2016, we made a patent application on the use of poly(hydroxy)oxetanes as hot-melt adhesives in wood bonding [34]. This study presents the synthesis and molecular characteristics of poly-(3-ethyl-3-hydroxymethyl)oxetanes of different molar masses and their adhesive interactions with polar substrates. According to our best knowledge, the present study is the first to discuss POX–lignocellulosic adhesive interactions.

In our approach, POXs were synthesized from 3-ethyl-3-(hydroxymethyl)oxetane by using 1,1,1-tris(hydroxymethyl)propane (TMP) as a core molecule. Such protocol allows for the yield of polymers of a lower dispersity when compared to homopolymerization and easier molar mass control [35].

## 2. Materials and Methods

All chemicals were purchased from Sigma-Aldrich (Poznań, Poland) and used as received; 3-ethyl-3-(hydroxymethyl)oxetane (EHO) was purchased from TCI Europe N.V., (Zwijndrecht, The Netherlands), and used as obtained. Solvents were distilled prior to use.

### 2.1. NMR and FTIR Measurements

^1^H and ^13^C NMR spectra were recorded on a Varian Mercury VXR 400 MHz (Agilent Technologies, Santa Clara, CA, USA) NMR spectrometer and used tetramethylsilane as an internal standard in DMSO-d_6_. FTIR spectra were recorded on a Nicolet iS5 (Thermo Fisher Scientific, Inc., Waltham, MA, USA) spectrometer in attenuated total reflectance (ATR) mode. The measurements were performed in the 400–4000 cm^−1^ range with a resolution of 2 cm^−1^.

### 2.2. Gel Permeation Chromatography (GPC)

The molar mass and molar mass distribution of the samples of polymers were performed via gel permeation chromatography (GPC) on a Viscotek (Malvern Panalytical Ltd., Malvern, UK) system comprising a GPCmax and a triple detector array (TDA) 305 unit equipped with one guard and two divinylbenzene (DVB) Jordi (JordiLabs LLC., Mansfield, MA, USA) gel columns (102–107; linear; mix bed) in methylene chloride as an eluent at 35 °C at a flow rate of 1.0 mL/min while using a refractive index (RI) detector and polystyrene calibration.

### 2.3. MALDI-TOF Spectrometry

The structure and morphology of the poly(hydroxy)oxetanes were confirmed in MALDI-TOF experiments. Spectra were measured on a Bruker UltrafleXtreme^TM^ (Bremen, Germany) instrument and used DCTB (trans-2-[3-(4-tert-butylphenyl)-2-methyl-2-propenylidene]malononitrile) as a matrix. Samples were dissolved in THF.

### 2.4. Lap Shear Strength

A 0.5 mm thick foil of a POX was applied between two 1.5 mm-thick birch veneers (120 × 20 mm) onto an overlapping area of dimensions 20 × 20 mm and bonded in a hot press at 150 °C for 30 s under 0.8 MPa pressure before immediately being transferred to a cold press and kept 5 min under 0.8 MPa pressure to cool down and set the bond-line. Bonded specimens were conditioned at normal conditions (20 ± 2 °C and 65 ± 5% relative humidity) for 24 h before testing (Instron 3369 universal testing machine; Instron Corp., Norwood, MA, USA). Twelve specimens were tested in each series. 

Shear strength (*R_t_*) was calculated from the Equation (1):

(1) $$R_{t}=\frac{F_{\max}}{S}$$

where *F_max_* is the maximum force in Newtons and *s* is lap area in mm^2^.

### 2.5. Contact Angle (θ) and Work of Adhesion (W_a_)

Wetting experiments were performed on a Phoenix 300 contact angle analyzer (Surface Electro Optics, Suwon City, Korea). Distilled water and diiodomethane were used as the reference liquids. The contact angles measurements were done 30 s after droplet deposition. Calculations of surface free energy were based on the Owens–Wendt method [36]. The work of adhesion between water and POX films was calculated from the Equation (2) [36]:

*W_a_* = *γ_S_* + *γ_L_* − *γ_SL_*, (2)

where *γ*_S_ is the surface energy of solid, *γ*_L_ is the surface tension of liquid, and *γ*_SL_ is the interfacial tension between a solid and a liquid (Equation (3)):

(3) $$\gamma_{SL}=\gamma_{S}+\gamma_{L}-2\sqrt{\gamma_{S}^{D}\gamma_{L}^{D}-2\sqrt{\gamma_{S}^{P}\gamma_{L}^{P}}},$$

where *D* and *P* denote the dispersive and polar parts of the free surface energy, respectively. Values for reference liquids were given elsewhere [37].

### 2.6. Synthetic Procedures

Cationic polymerization of 3-ethyl-3-(hydroxymethyl)oxetane (EHO) with 1,1,1-tris(hydroxymethyl)propane (TMP):

The poly(hydroxy)oxetanes (POXs) were prepared according to the following procedure: in a 250 mL three-neck flask equipped with a magnetic stirrer, thermometer, a rubber septum, a funnel, a nitrogen inlet and a bubble meter, 300 mL of dichloromethane and 2.36 g (17.62 mmol) of TMP were placed. The reaction vessel was degassed by using nitrogen for 20 min, and then boron trifluoride diethyl etherate BF_3_Et_2_O (0.13 g, 0.92 mmol) was added via syringe and heated to 70 °C. Then, 10.25 g (88.36 mmol) of EHO was added dropwise at a rate of 5 mL/h. After 2 h at 70 °C, the reaction was quenched with ethanol. The product was precipitated in cold diethyl ether and dried under vacuum.

POXs of various TMP/EHO ratios, as well as a POX without TMP core, were prepared according to the same procedure. Products were obtained with yields ranging between 89% and 95%.

## 3. Results and Discussion

**Figure 1:**
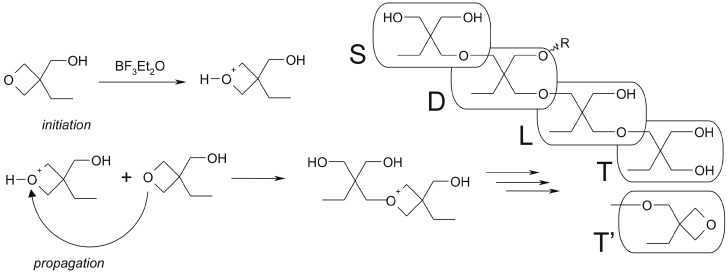
Cationic polymerization of 3-ethyl-3-hydroxymethyloxetane.

**Figure 2:**
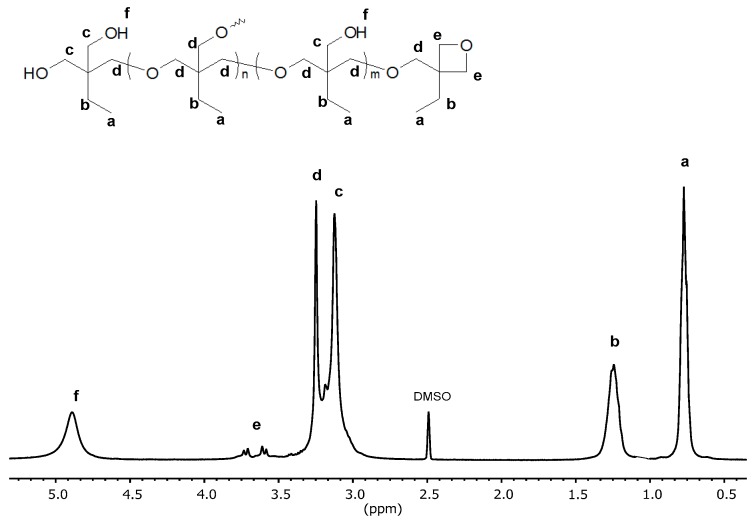
^1^H NMR (400 MHz, DMSO-d_6_) spectrum of poly(3-ethyl-3-hydroxymethyloxetane).

Poly(3-ethyl-3-hydroxymethyloxetane)s can be synthesized according to cationic, anionic or activated monomer mechanisms [1,2,13,15,38,39]. In the case of cationic polymerization, the catalyst concentration and temperature have fundamental impacts on the degree of the branching of the obtained product. Figure 1 shows the mechanism of the reaction and the possible substructures of the polymer. In the structure that was synthesized with the use of trimethylolpropane (TMP) as an initiator and EHO as a monomer, the starting (S) and terminal (T) units were identical, which simplified the NMR spectra (Figure 2). Other units present in the polymer were dendritic ones (D) in which all hydroxyl groups reacted; linear ones (L), with one free OH group; and terminal cyclic ones (T’).

In case of the polymerization of 3-ethyl-3-hydroxymethyloxetane at a constant concentration of catalyst, the number of branching points depended mainly on the reaction temperature. When the reaction was conducted at −30, −2, 30 and 70 °C, the degrees of branching calculated from ^13^C NMR spectra by using the Frey equation [38] were equal to 0.21, 0.36, 0.43 and 0.50, respectively.

Figure 2 shows a typical ^1^H NMR spectrum of hyperbranched poly(3-ethyl-3-hydroxymethyloxetane) measured in DMSO-d_6_. All the signals can be assigned to the structural elements of the polymer: –OH (4.7 ppm), −CH_2_−O−(T’) (3.7–3.6 ppm), −CH_2_−O−(3.3 ppm), −CH_2_−OH (3.1 ppm), −CH_2_−CH_3_ (1.3 ppm), and −CH_2_−CH_3_ (0.8 ppm). The doublet of doublets (e) at 3.7 ppm indicates the presence of terminal units T’ (Figure 1) and was visible only for the polymers synthesized at low temperatures. 

**Figure 3:**
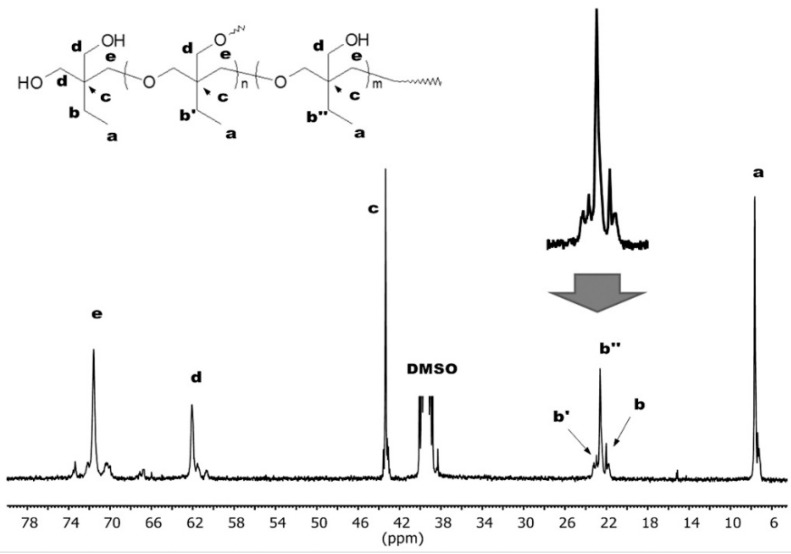
^13^C NMR (400 MHz, DMSO-d_6_) spectrum of poly(3-ethyl-3-hydroxymethyloxetane) of degree of branching equal to 0.36.

The degree of branching of the hyperbranched polyoxetanes was determined with the use of b signals (Figure 3) in the ^13^C NMR spectrum. The signal of the methylene group carbon of the ethyl substituent was split according to the chemical surrounding into signals of dendritic (b’’), linear (b’) and terminal (b) units. It was deconvoluted and integrated by using MestReNova 10.0.2-15465 software (Mestrelab Research, S.L., Escondido, CA, USA).

For the purpose of adhesive tests, four polyoxetanes of different molar masses were synthesized. Reagent amounts and theoretical molar masses are shown in Table 1. A high abundancy of polar hydroxyl groups is a prerequisite for an adhesive material to form strong interactions with polar substrates. Additionally, thermoplastic character of POXs makes them considered to be potential hot-melt adhesives.

**Figure 4:**
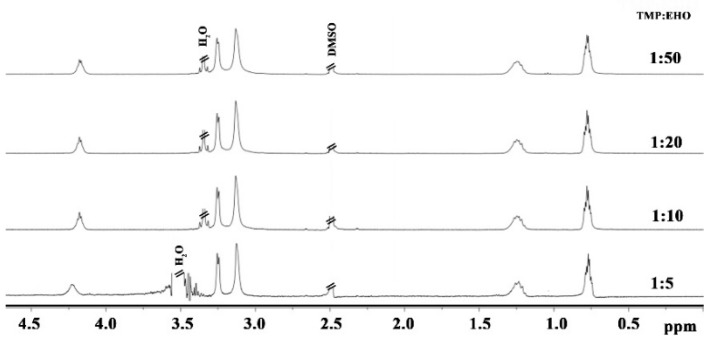
^1^H NMR (400 MHz, DMSO-d_6_) spectra of the studied polyoxetanes.

The reactions were performed at 70 °C to assure high (0.5) degrees of branching and high (>95%) reaction yields. The ^1^H NMR spectra shown in Figure 4 indicate the efficient polymerization of the EHO monomer with the TMP core molecule. The signals of TMP units have the same chemical shifts as signals coming from EHO units which make the spectra quite simple. For the same reason, the calculation of molar mass of the polymers from the NMR spectra was not possible. The T’ units signals in these experiments were not visible.

**Figure 5:**
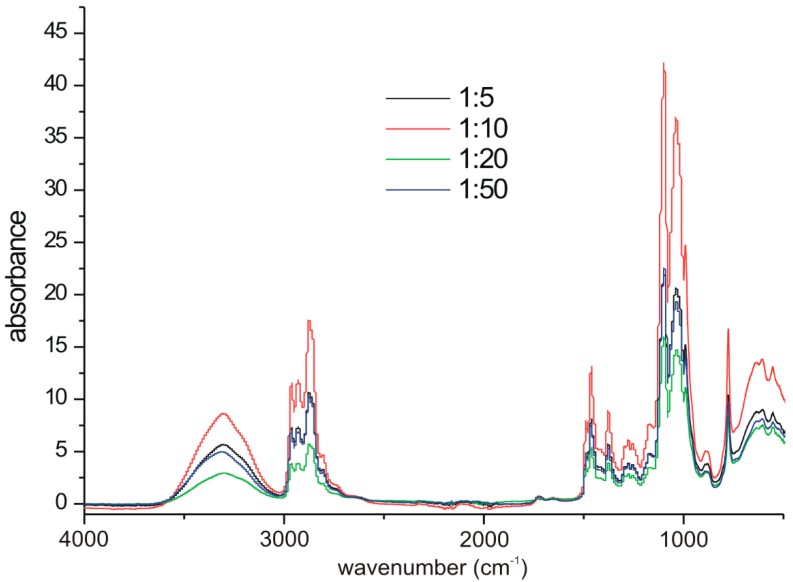
FTIR spectra of poly(3-ethyl-3-(hydroxymethyl)oxetanes).

In the FTIR spectra presented in Figure 5, O–H stretch (3324 cm^−1^), CH_2_ stretch (2966 cm^−1^, 2934 cm^−1^ and 2880 cm^−1^) and backbone ether C–O–C stretch (1180 cm^−1^ and 1047 cm^−1^) bands are present. It is known that isolated OH groups appear at the ca. 3600 cm^−1^ wavenumber, whereas hydrogen-bonded hydroxyls appear at lower wavenumbers (3400–3200 cm^−1^) [40]. Thus, the band observed at 3324 cm^−1^ proved the presence of intra- and intermolecular hydrogen bonds within the POXs. Mamiński et al. demonstrated that the partial substitution of hydroxyls in a hyperbranched polymer reduces intramolecular hydrogen-bond formation [41].

No relationship between the reagents ratio in the POX and peak intensities could be seen, because the FTIR measurements were qualitative. On the other hand, it is clear that respective bands in the spectra of 1:5–1:50 occurred at identical wavenumbers, thus proving great similarity in their structures.

**Figure 6:**
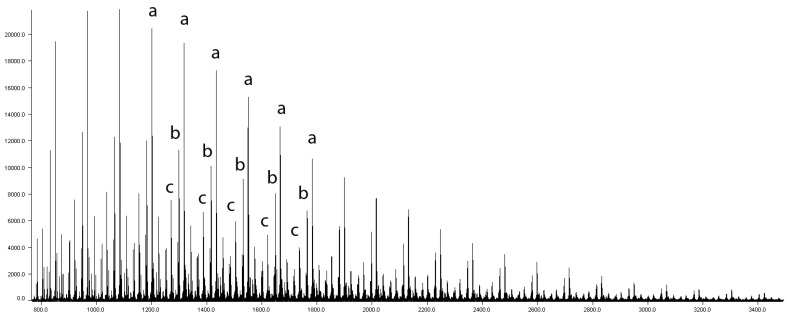
MALDI-TOF (DCTB, Na+) spectrum of POX with 1,1,1-tris(hydroxymethyl)propane (TMP) core; a-a distance *Δm/z* = 116.

The MALDI-TOF technique allows for a deeper insight into the morphologies and microstructures of polymers. It allows for the confirmation of the mass of repeating unit and the residual mass related to the end group or core molecule. A detailed analysis of the structure of the obtained POXs was performed with this technique. An exemplary MALDI-TOF spectrum is shown in Figure 6. The distance between the a–a (b–b or c–c) peaks was equal to 116 *m/z*, which corresponds to a repeating unit of poly(3-ethyl-3-(hydroxymethyl)oxetane). All signals were observed as sodium cation adducts. The most intensive series a can be ascribed to the molecules with the TMP core. The b series *m/z* values were lower by 18 and can be ascribed to the macromolecules that contained one cyclic structure T’ formed via the dehydration of the terminal T structure and cyclization (Figure 1) [2]. As the reaction was quenched with ethanol, the c series came from POX macromolecule end-capped with ethoxy group. Other signals were in the minority and may have come from products of water elimination.

Theoretical macromolecule weight and size are determined by the amounts and structure of used reagents—the monomer to core ratio. In practice, thstatistical nature of polymerization yields a mixture of products of various shapes, branching degrees and molar masses [42]. The molar mass and dispersity of the synthesized polymers are presented in Table 2. One can see that dispersity grew with the growth of the TMP/EHO ratio. This is in agreement with the nature of the ring-opening multibranch polymerization reaction [14,43]. The table does not show a clear relationship between the theoretical and observed values of the number-average (*M*_n_) and weight-average (*M*_w_) molar mass. This is not surprising because the GPC technique is considered to be error-burden when using polystyrene as the internal standard, because polystyrene’s hydrodynamic radius is different from that of the POX. Difficulties in the reliable determination of true average molar mass of hyperbranched polymers by GPC have been described in the literature [44,45,46].

It is commonly agreed that the adhesive interactions of a liquid with a substrate are determined by the surface free energy of the phases, as well as by the interfacial tension between them [36]. It is apparent from Equation (3) that when the interfacial tension, *γ_SL_*_,_ is equal to zero, the work of adhesion is maximized. The data in Table 3 indicate that water contact angle (*θ*) on the POX film (64–67°) was lower than that for the reference commercial hot melt adhesive (82°) and lower than that of polyurethane adhesives (75°) [47], which resulted from the highly polar character of POX due to a high abundancy of hydroxyl groups; this low water contact angle demonstrates strong adhesive interactions with polar substrates. The supposition is confirmed by the work of adhesion (*W_a_*) values 101–105 mJ/m^2^, which were just ca. 20% lower than the work of adhesion of water on natural wood surfaces (126–133 mJ/m^2^) [36]. Thus, the abovementioned surface characteristics of POXs’ films are a sufficient ground to hypothesize that POXs are efficient hot melt adhesives for polar substrate bonding.

**Figure 7:**
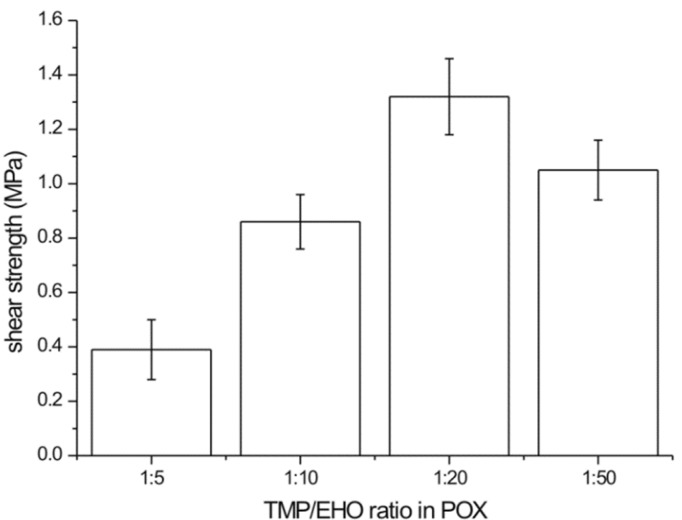
Shear strengths of the POX bond-lines.

**Figure 8:**
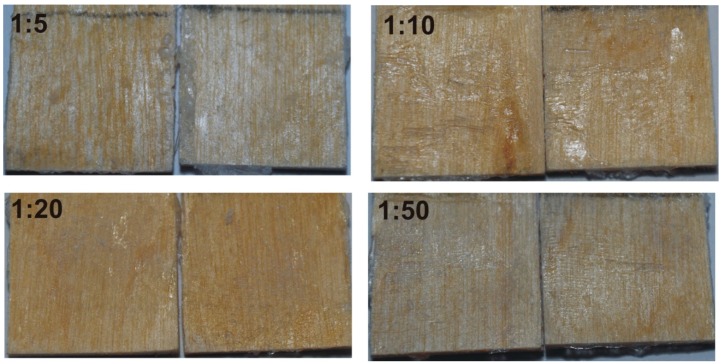
Cohesive fracture in POXs layers of different TMP/EHO (3-ethyl-3-(hydroxymethyl)oxetane) ratios.

**Figure 9:**
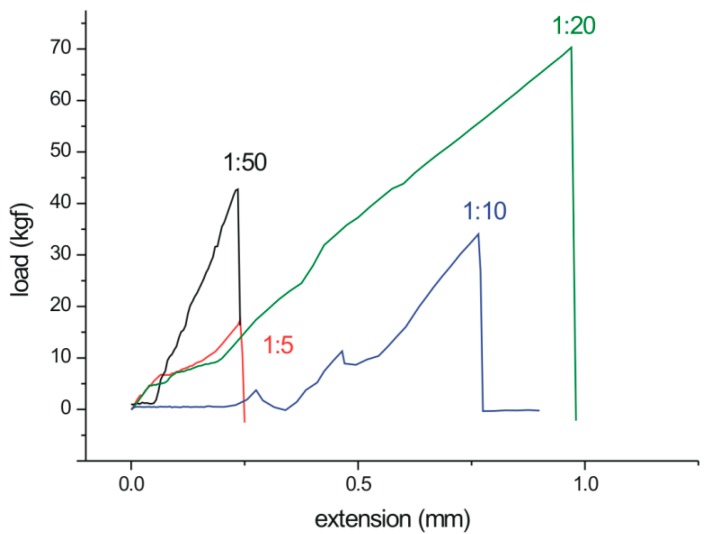
The effect of the reagent ratio in the POX molecule on load bearing ability.

In order to empirically verify the hypothesis, the softening and flow points of the polymers were determined (Table 3). The *T*s values were comparable to those of the commercial hot melt adhesives (88–105 °C) used in furniture manufacturing. Bonding experiments performed on birch veneers revealed apparent differences in joints shear strength (Figure 7). One can see that the POX 1:20 and 1:50 series exhibited much higher values (1.32 MPa and 1.05 MPa, respectively) than those of the 1:5 and 1:10 series (0.39 MPa and 0.86 MPa, respectively). The phenomenon is associated with both the abundancy of hydroxyl groups and the ability of hydrogen bond formation, which increases with the degree of branching as well as with the mechanical properties of POXs. Neither of them exhibited cohesive failures in wood, whereas cohesive failure in the POX layer was found in each case (Figure 8). Such behavior resulted from an adhesion to polar substrate that was higher than the cohesion within POXs. As shown in Figure 9, in all cases, a brittle fracture occurred at a low extension during the shear test (0.18–0.97 mm). It is known from the literature that brittle materials fracture under relatively low stress [48], and, moreover, the performance of high modulus adhesives on low modulus substrates is not optimal [49]. However, the brittle character of the investigated POXs cannot be associated with a highly crystalline structure since Mai et al. demonstrated that the degree of crystallinity decreases with increasing degree of branching in POX and is close to 0% for the degree of branching ca. 40% [11]. The highest extension to fracture was observed for the 1:20 POX, which indicates its abilities for higher deformation and stress dissipation than the other investigated POXs. Subsequently, the increased shear strength of the bond-line was yielded.

The mechanical properties of the bond-lines confirmed our assumptions regarding the strong adhesive interactions of POXs with polar substrates. It is noteworthy that the observed shear strength of the 1:20 series exceeded the minimum required for plywood (≥1.0 MPa) [50] and were comparable to those reported for poly(lactide)-poly(ε-caprolactone)-based and ethylene-vinyl acetate based hot-melt adhesives (0.6–1.5 MPa [51]) but still lower than those reported for FeCl_3_-curable polyoxetanes [30]. Bekhta and Sedliačik investigated plywood bonding with HDPE and demonstrated that shear strengths of the bond-lines greatly depended on the time, pressure, and temperature of the process [52]. The authors obtained the highest strengths for a 160 °C temperature and a 3 min press time, which were necessary for proper penetration of the molten adhesive into the substrate. Similar observations were made by Kajaks and co-workers for polypropylene-based hot-melts [53]. As it is commonly agreed that the quality of bonding strongly depends on processing parameters such as temperature, time and pressure [54], further studies on POX bonding parameters are required. Keeping in mind that the optimization of bonding quality was not the objective of this study, it is likely that a prolonged pressing time and increased temperature would result in the improved strengths of bond-lines.

Thus, the studied POX-based adhesives seem plausible to be used where high mechanical properties of adhesives are not required, e.g., packaging or veneering applications.

However, these findings also indicate that that further research should be aimed at the detailed characterization of the thermal, rheological and mechanical properties of POXs, as well as their enhancement via grafting, filling or blending, so that an improved mechanical performance and stress dissipation ability can be incorporated into polymers.

## 4. Conclusions

Hyperbranched poly(hydroxyl)oxetanes (POXs) were synthesized from 1,1,1-tris(hydroxymethyl)propane (TMP) as a core molecule and 3-ethyl-3-(hydroxymethyl)oxetane (EHO) as a branching monomer. Their hyperbranched structure and chain morphology were confirmed in NMR and MALDI-TOF experiments. It was shown that dispersity grows with the growth of the TMP/EHO ratio, which remains in agreement with the nature of the ring-opening multibranch polymerization reaction. The thermoplastic character of the polymers predisposes them to be potential hot melt adhesives. An analysis of POXs behavior in contact with polar materials proved: (1) strong interactions with polar substrates and high work of adhesion; (2) adhesive interactions with wood that were higher than cohesion within the polymer; (3) the effect of macromolecule structure on the tensile shear strength in the bond-line; and (4) a brittle fracture mode in bond-line.

These findings indicate the need for further investigations on the thermomechanical performance and enhancement of the mechanical properties of poly(hydroxy)oxetanes via chemical modification or doping. Our studies will continue.

## Figures and Tables

**Table 1:** Molar mass and amounts of reagents used in synthesis of polyoxetanes (POXs).

Theoretical Molar Mass [g/mol]	TMP/EHO Molar Ratio	EHO [g]	TMP [g]	BF_3_Et_2_O [g]
714	1:5	10.25	2.36	0.13
1295	1:10	18.00	2.36	0.25
2457	1:20	20.50	1.18	0.25
5942	1:50	51.58	1.18	0.63

**Table 2:** Molar mass and dispersity (D) of POXs determined by gel permeation chromatography (GPC).

TMP/EHO Molar Ratio	*M*_n_	*M*_w_	*D*
1:5	1240	1690	1.77
1:10	1220	1650	2.18
1:20	1160	1390	2.52
1:50	1310	1850	3.57

**Table 3:** Selected thermal and surface properties of the studied POXs.

TMP/EHO Molar Ratio	*T*_s_ [°C]	*T*_f_ [°C]	*θ* [deg]	*W*_a_ [mJ/m^2^]
1:5	88	105.1	67.2 ± 4.6	101.3 ± 7.2
1:10	87	105.4	66.5 ± 0.6	102.2 ± 0.7
1:20	88	105.2	64.1 ± 2.9	104.5 ± 3.5
1:50	80	105.1	65.4 ± 1.0	104.8 ± 0.4
Reference ^a^	–	–	82.1 ± 4.4	80.3 ± 3.5

^a^ commercial hot melt; *T*_s_—softening point; *T*_f_—flow point; *θ*—water contact angle; *W_a_*—work of adhesion.
